# Understanding the Needs of Young and Middle‐Aged Chinese People Who Have Experienced a Stroke Who Have Not Successfully Returned to Work: A Qualitative Study

**DOI:** 10.1111/hex.70150

**Published:** 2025-01-15

**Authors:** Ziwei Liu, Shu Liu, Jiaxing Shi, Yanming Yang, Yuan Zhong, Jiaxin Li

**Affiliations:** ^1^ Nursing Department The First Affiliated Hospital of Henan University of Traditional Chinese Medicine Zheng Zhou He Nan China; ^2^ The Fifth Affiliated Hospital of Sun Yat‐Sen University Zhu Hai He Nan China; ^3^ College of Nursing Xinyang Vocational and Technical College Xin Yang Henan China

**Keywords:** phenomenological study, qualitative research, return to work, Stroke, young and middle‐aged adults

## Abstract

**Objectives:**

The study aims to understand the return to work (RTW) needs of young and middle‐aged people who have experienced a stroke and to contribute to the development of supportive RTW services.

**Design:**

A qualitative study employing the phenomenological method.

**Participants:**

Eleven young and middle‐aged people who have experienced a stroke participated in the study.

**Methods:**

Semi‐structured in‐depth interviews were conducted and analysed using Colaizzi's 7‐step method to identify and categorize the RTW needs of participants.

**Results:**

The analysis delineated four overarching thematic categories of RTW needs among the participants: self‐management needs, emphasizing the regulation of symptoms, health maintenance and recovery planning; social support needs, highlighting the significance of workplace accommodations, professional medical guidance, and emotional encouragement from companions and family; the need for information related to returning to work, which includes accessing resources on rehabilitation opportunities, labour rights and professional consultation services; and personal development needs, focusing on fostering self‐worth, identifying growth opportunities and acquiring new skills to adapt to changing professional demands.

**Conclusion:**

The diverse and comprehensive needs of young and middle‐aged people who have experienced a stroke underscore the importance of multifaceted support from healthcare professionals. This support should encompass medical, psychological, informational and skill‐development aspects and should involve enhanced communication and collaboration with relevant stakeholders to facilitate a successful RTW.

**Patient or Public Contribution:**

This study was designed without direct involvement from patients or the public in the development of the research question, the design of the study, or the conduct of the research. This decision was informed by the specific focus on qualitative experiences and perceptions of stroke survivors regarding their RTW journey, which relied heavily on personal narratives and subjective accounts collected through individual interviews. However, the insights gained from these narratives have been crucial in shaping the research outcomes, emphasizing the patient‐centred approach to understanding RTW barriers and facilitators.

**Reporting Method:**

This study followed the SRQR checklist for qualitative studies as its reporting method.

## 1 Introduction

Stroke remains the primary cause of death and disability worldwide, with its burden increasingly affecting developing countries [1]. Data from 2021 indicate that the prevalence of stroke among young and middle‐aged individuals has escalated to 40% [2] and nearly one‐third of all strokes now occur in adults of working age [3]. In China, stroke constitutes a significant chronic non‐communicable disease (NCD) that poses a severe threat to national health. According to the most recent Global Burden of Disease Study (GBD), China has the world's highest lifetime stroke risk at 39.9%, with an onset age lower than that in developed countries (65–75 years), affecting up to 15 million survivors. This younger onset age and the high number of survivors indicate a substantial stroke risk, placing China at an intermediate level internationally [4, 5]. Additionally, the incidence of stroke is rising, and the onset age is decreasing, as evidenced by 2020 data showing 49.2% of stroke cases occurring among those aged 40–59 years [6]. Post‐stroke residual symptoms, including functional, cognitive and psychological impairments, significantly challenge the affected individuals' work capabilities and quality of life [7]. Stroke's impact is particularly detrimental to young and middle‐aged adults, who are crucial to the nation's workforce; the high unemployment rates among these stroke survivors not only increase the burden on families and healthcare systems but also hinder societal progress [8].

Franche et al. [9] introduced the concept of return to work (RTW) in 2007, which involves the reintegration of an ill individual into their original or a new job, either part‐time or full‐time. Timely failure to RTW post‐stroke not only impedes the patient's recovery process but also diminishes socialization and may induce negative emotions such as anxiety and depression [10]. RTW has been recognized as a significant indicator of recovery [11], and delays in this process can reduce a patient's willingness to RTW over time [12]. Furthermore, research indicates that up to 84% of patients report unmet needs regarding RTW 2 years post‐stroke [13]. Thus, early identification of the factors influencing RTW and the needs associated with it is crucial for enhancing the quality of life and social participation of stroke survivors.

Current research predominantly focuses on the disease‐related needs of young and middle‐aged people who have experienced a stroke [14, 15]. Although previous qualitative studies have examined the lived experiences of patients returning to work and identified barriers and facilitators to work participation [16, 17, 18], the RTW process for stroke survivors is multifaceted and extends beyond these elements alone. Notably, the role of environmental factors—such as workplace conditions, healthcare professionals, social welfare systems, legal aspects and support from family and friends—remains underexplored, especially concerning the expectations and needs of young and middle‐aged stroke survivors regarding RTW. One study summarizing the unmet care needs of stroke survivors found that the proportion of needs related to employment ranged from 6.9% to 59.6% [19], highlighting a significant demand for supportive services from healthcare professionals concerning RTW issues. Recognizing these needs and expectations is crucial for developing effective intervention programmes. Therefore, this study aims to conduct in‐depth interviews with young and middle‐aged stroke survivors to better understand their RTW needs, providing both theoretical and practical foundations for future tool development and intervention research.

## 2 Methods

### 2.1 Study Design

This study employed a qualitative design, utilizing descriptive phenomenological methods to explore the lived experiences of people who have experienced a stroke in relation to their RTW process. Data were collected through semi‐structured interviews, enabling an in‐depth exploration while allowing participants the flexibility to comprehensively express their perspectives.

### 2.2 Ethical Considerations

This study was approved by the Ethics Committee of Henan University School of Nursing and Health (HUSOM2021‐288) and conducted in strict adherence to ethical guidelines and regulations, ensuring research integrity and participant rights.

### 2.3 Sampling Strategy

This study employed purposive sampling to recruit young and middle‐aged stroke patients in the recovery phase from three communities in Zhengzhou City between December 2022 and July 2023. To ensure diversity, a maximum variation sampling strategy was also applied. According to WHO guidelines, stroke survivors with stable vital signs, consciousness and nonprogressive conditions are eligible for early rehabilitation, typically starting 10–14 days posthemorrhagic stroke. For this study, based on literature and expert consultations, the rehabilitation period was defined as ≥ 6 weeks from the most recent stroke event.

### 2.4 Participant Selection

A purposive sampling method was used to select young and middle‐aged people who had experienced a stroke from three communities in Zhengzhou City, all of whom were in the rehabilitation period. A maximum variation sampling strategy was adopted to ensure a comprehensive and enriched understanding of the RTW needs of young and middle‐aged stroke survivors. Inclusion criteria are (i) age range from 18 to 59 years, classified as young and middle‐aged adults [20]; (ii) patients diagnosed with stroke confirmed by cranial CT or magnetic resonance imaging [21]; (iii) patients employed before the stroke who have not yet successfully returned to work; (iv) patients with adequate communication skills who voluntarily agree to participate in the interview. Patients who were unable to communicate or had other severe comorbidities were considered ineligible for inclusion in the study.

### 2.5 Data Collection

The interviewer served as the primary research instrument, utilizing audio recording equipment for documentation. The interview outline was developed based on a review of domestic and international literature and consultations with experts in cerebrovascular nursing. The questions included the following: (1) What does work mean to you? (2) What do you think is the impact of the stroke on your ability to work? (3) What factors do you believe will affect your RTW? (4) Do you need support in preparing for RTW? (5) What expectations and needs do you have from individuals, family, healthcare organizations or society? (6) What kind of support (informational, psychological, rehabilitative) would you like to receive, and from whom, in preparation for your RTW? What does work mean to you?

Face‐to‐face semi‐structured interviews were conducted by two experienced researchers, each with over 10 years of expertise. Interviews, lasting 25–40 min, were held in hospital interview rooms or participants' homes. Recruitment was facilitated through study information posted on a community bulletin board, with interested participants contacting the researcher via phone or WeChat. Eligibility was assessed, and those meeting the criteria received an information sheet detailing the study's purpose, procedures and confidentiality assurances, with voluntary consent obtained. Interviews were flexible, guided by participant responses, and enriched by observations of non‐verbal cues. Audio recordings ensured accuracy. Data collection continued until meaning saturation was reached, defined as no new themes emerging from additional interviews, aligning with Hennink et al.'s recommendations [22]. This iterative approach ensured a thorough understanding of the study's themes.

### 2.6 Data Collation and Analysis

Interview data were transcribed verbatim within 24 h and double‐coded by two independent researchers to ensure reliability. Using Colaizzi's seven‐step approach [23], the data were repeatedly reviewed to extract meaningful statements and generate codes. Core themes were synthesized by integrating patients' statements and identifying connections between themes. Credibility was enhanced through iterative comparison of coded data with original recordings and validation by some participants, incorporating their feedback to refine findings. Ambiguities were resolved through group discussions, ensuring consensus and consistency in the final results [24].

### 2.7 Rigour

To ensure study rigour, interviews were conducted by two experts with over ten years of interviewing experience, while two‐stroke nursing specialists coded and analysed the data. All researchers contributed to developing the interview guide and refining coding results. Credibility was enhanced through member checking, allowing participants to validate identified themes and subthemes. Group discussions facilitated reflection on interview conduct and data analysis approaches. Additionally, all researchers received rigorous training in qualitative research methodologies, ensuring reliable execution and valid results.

## 3 Results

### 3.1 General Information of Interview Subjects

The study included a total of eleven people who have experienced a stroke, comprising eight males and three females, with a mean age of 43.27 ± 7.79 years. Most participants had attained a high school education or higher, and the majority were married. The demographic details of the study population are summarized in Table 1.

**Table 1:** General information about the study population.

Code	Sex	Age	Educational level	Marital status	Residency	Monthly salary	Stroke type	Career	Functional impairment	Years since stroke
S1	Female	38	Junior college	Married	Town	3000–5000	Cerebral infarction	Office worker	Yes	≤ 1 year
S2	Male	54	Primary school	Married	Rural	1000–3000	Cerebral infarction	Fitter	Yes	> 3 years
S3	Male	47	Middle school	Married	Town	1000–3000	Cerebral haemorrhage	Skilled worker	Yes	1–3 years
S4	Female	52	Primary school	Married	Rural	1000–3000	Cerebral infarction	Janitorial staff	No	> 3 years
S5	Female	28	Undergraduate	Unmarried	Town	＞ 5000	Cerebral infarction	Self‐media worker	Yes	1–3 years
S6	Male	50	Middle school	Married	Town	3000–5000	Cerebral haemorrhage	Teacher	Yes	> 3 years
S7	Male	46	Middle school	Married	Rural	1000–3000	Cerebral infarction	Workman	No	1–3 years
S8	Male	43	High school	Married	Rural	3000–5000	Cerebral infarction	Self‐employed	No	1–3 years
S9	Male	39	High school	Married	Town	＞ 5000	Cerebral haemorrhage	Lorry driver	Yes	≤ 1 year
S10	Male	44	Junior college	Married	Town	3000–5000	Cerebral infarction	Functionary	Yes	> 3 years
S11	Male	35	Junior college	Married	Town	3000–5000	Cerebral infarction	Salesman	Yes	≤ 1 year

### 3.2 Interview Data Analysis Results

**Figure 1:**
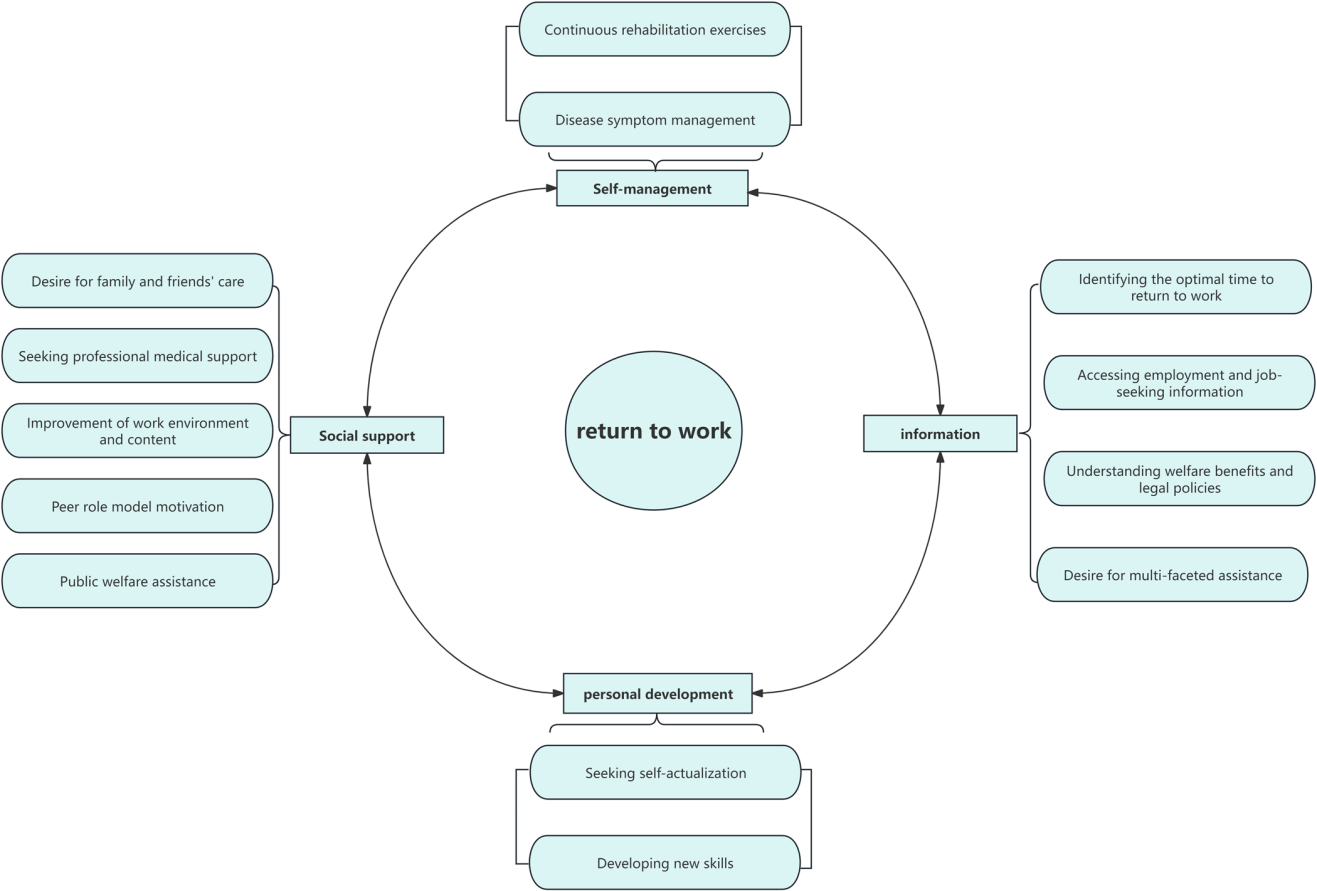
Framework of interview themes.

The researchers conducted semi‐structured interviews with 11 young and middle‐aged people who had experienced a stroke. They organized, coded and extracted meaningful statements from the interview data to identify themes. Based on the results, the researchers summarized four main themes and 13 sub‐themes regarding the work return needs and expectations of these patients: (1) self‐management needs; (2) social support needs; (3) need for information related to returning to work; (4) personal development needs. The coding process and thematic framework diagram are shown in Table 2 and Figure 1, respectively.

**Table 2:** Coding Process for Interview Findings.

Main theme	Subtheme	Theme prototype	Representative quote
Self‐management needs	Management of disease symptoms	Patients' needs for managing post‐stroke residual symptoms, such as functional impairments, fatigue and memory decline, which hinder their return‐to‐work readiness.	S1: Physical mobility is not as good as it used to be, I feel tired when I stand for a little bit longer, not to mention going back to work.
	Continuation of rehabilitation exercises	Patients' concerns about the lack of continuity in rehabilitation services after discharge, affecting their physical recovery and quality of life.	S2: Rehabilitation ended too soon, if I could have done more physical therapy for a while I think I would have recovered better.
Social support needs	Desire for care from family and friends	Patients' desire for emotional support and encouragement from family and friends to boost their confidence in returning to work.	S5: After I got sick, my friends often called me to encourage me, telling me that I am not an invalid, and that there are many things I can do, and listing what I can do, which makes me feel much better.
	Seeking professional medical support	Patients' expectations for healthcare professionals to provide not only medical care but also psychological and work‐related advice.	S4: I can't say a few words to the doctors and nurses every day, and I have less time to communicate with them. Sometimes I want to ask them something and see that they are busy, but I don't feel like bothering them.
	Improvement of work environment and content	Patients hope employers can modify work environments or provide assistive tools tailored to their physical limitations.	S6: I don't think I'll be able to do much physically when I go back to work, so I definitely won't be able to be a classroom teacher, and I'll need to adjust my workload and time.
	Motivation from peer role models	Patients seek inspiration and practical advice from peers who have successfully returned to work after a stroke.	S3: If there are any patients who have already managed to find a job after a stroke, they can teach us a little bit about their experiences and share with each other.
	Public welfare assistance	Patients hope for financial aid or other forms of public support to alleviate the economic burden caused by stroke.	S9: The unit dismissed me directly, my daughter is still studying in secondary school, there are still a lot of places where I need money, I see that there seems to be some kind of unemployment benefits and sickness benefits in foreign countries, it would be good if we also have them here.
Need for information related to returning to work	Identifying the optimal time to return to work	Patients seek professional guidance on when they can safely and effectively return to work based on their recovery progress.	S9: Doctors usually give advice mainly on body diet and so on, without mentioning about work, so that means I'm not suitable to go to work at this stage either, right?
	Accessing employment and job‐seeking information	Patients want to explore suitable job opportunities but lack effective ways to access relevant information.	S7: I don't know how to look for a job on the Internet. As I get older, I find it difficult to use my smartphone.
	Understanding welfare benefits and legal policies	Patients desire to learn more about welfare policies and labour laws to protect their rights during the return‐to‐work process.	S10: I'm still considered mid‐level leadership in my unit. Before my illness, the unit had me do my best in my role. However, after my illness, the leadership used my health as a reason to persuade me to move to a non‐leadership position. But I don't have the heart to care about this now.
	Desire for multi‐faceted assistance	Patients want co‐operation between different entities such as healthcare providers, employers and social agencies to facilitate their return to work.	S11: I hope the state can improve laws and regulations to make it mandatory that employers are not allowed to discriminate against job seekers who have been sick.
Personal development needs	Pursuit of self‐actualization	Patients view returning to work as a means to regain their sense of self‐worth and social identity.	S6: In addition to being a means of earning a living, work helps me continue learning new knowledge, keep up with societal developments, and stay connected with society.
	Need for Developing New Skills	Patients hope to acquire new skills to meet the demands of potential job opportunities after a stroke.	S9: I used to drive a truck, but I can't drive anymore after I got sick. Now, at my age, it's hard to find a job, and I don't know what to study anymore.

### 3.3 Self‐Management Needs

#### 3.3.1 Management of Disease Symptoms

Patients' readiness to RTW is hampered by residual health problems following a stroke, including dysfunction, fatigue, memory loss and psychological disillusionment. Limited mobility due to physical dysfunction is the most significant factor affecting their RTW, while mental fatigue and short‐term memory problems impair their ability to concentrate and perform. Additionally, some patients experience psychological loss after illness, marked by anxiety and frustration about the physical changes caused by their condition, which further diminishes their desire to RTW.

*S1: Physical mobility is not as good as it used to be, I feel tired when I stand for a little bit longer, not to mention going back to work.*

*S3: I feel like I have an arm that I can't use, and no one will want to use me, so how low is the efficiency of this work.*

*S8: After I got sick, I felt that my spirit was not very good, and I couldn't concentrate on things, and I couldn't get up the energy for anything.*

*S11: My memory has deteriorated, as if I have Alzheimer's disease, and I often don't remember where I put the things I just used here, so I am afraid of what I will do if I go back to work and often lose things.*

#### 3.3.2 Continuation of Rehabilitation Exercises

When patients transition from hospitalization to home care after a stroke, inconsistencies in their rehabilitation services can arise. The lack of duration and intensity in rehabilitation treatment, along with interruptions in subsequent medical rehabilitation services, can directly impact the process of physical recovery and the quality of life of these patients.

*S2: Rehabilitation ended too soon, if I could have done more physical therapy for a while I think I would have recovered better.*

*S5: It seems that there is not much care after discharge from the hospital, the rehabilitation therapist had follow up visits at the beginning, then regular follow up visits were not available.*

*S8: Only in the hospital can I do the rehabilitation exercise on time, after I was discharged from the hospital, firstly, I don't have those rehabilitation therapists any more, and secondly, no one supervises you any more, so I don't do it any more.*

### 3.4 Social Support Needs

#### 3.4.1 Desire for Care From Family and Friends

Patients reported that the concern and encouragement of family and friends played an important role in their recovery, helping to increase their confidence in returning to work.

*S2: I don't have many friends, and my family doesn't care much about me, so I still hope that I can have someone to talk to, and someone to talk to will make me feel better*.

*S5: After I got sick, my friends often called me to encourage me, telling me that I am not an invalid, and that there are many things I can do, and listing what I can do, which makes me feel much better.*

*S11: My parents are my biggest spiritual support. When I said I didn't have confidence in my old job, they said they would fully support me no matter what I did, so what reason do I have to give up on myself? I'm very glad I have them with me.*

#### 3.4.2 Seeking Professional Medical Support

Some patients stated that healthcare staff communicated too little with them and did not pay enough attention to their psychological needs and RTW concerns. They hoped that healthcare staff would communicate more patiently, listen to their demands and worries and provide suggestions and guidance on returning to work.

*S1: I talk to doctors and nurses every day about my illness, but I never discuss my work.*

*S4: I can't say a few words to the doctors and nurses every day, and I have less time to communicate with them. Sometimes I want to ask them something and see that they are busy, but I don't feel like bothering them.*

#### 3.4.3 Improvement of Work Environment and Content

Patients believe that returning to work requires adjustments to the work environment, work intensity and schedule. They hope employers can provide suitable assistive devices to help them complete their tasks according to their physical conditions.

*S1: If I go back to work, I may not be able to do a lot of things. I hope the company can give me a tape recorder or a work content manual.*

*S6: I don't think I'll be able to do much physically when I go back to work, so I definitely won't be able to be a classroom teacher, and I'll need to adjust my workload and time.*

*S10: I can't move my left hand very well, and it would be nice if the organization would provide me with a computer keyboard for one‐handed use.*

#### 3.4.4 Motivation From Peer Role Models

The incidence of stroke is increasingly affecting younger individuals. When returning to work, patients not only endure physical pain but also face significant mental pressure. Patients need to communicate regularly with fellow patients and share their experiences of returning to work. Seeing patients with similar conditions successfully RTW can be particularly encouraging. Through the power of these examples, patients can find internal motivation and rebuild the courage to RTW.

*S3: If there are any patients who have already managed to find a job after a stroke, they can teach us a little bit about their experiences and share with each other.*

*S5: It's quite hard to go it alone, it would definitely be much better if someone could share some information with each other.*

#### 3.4.5 Public Welfare Assistance

After a stroke, the combination of high medical bills and reduced income due to cessation of work imposes a significant financial burden on the family. Patients long for more external attention and assistance to help alleviate the burden of the disease.

*S5: I hope more people can pay attention to our group and help us.*

*S9: The unit dismissed me directly, my daughter is still studying in secondary school, there are still a lot of places where I need money, I see that there seems to be some kind of unemployment benefits and sickness benefits in foreign countries, it would be good if we also have them here.*

### 3.5 Need for Information Related to Returning to Work

#### 3.5.1 Identifying the Optimal Time to RTW

Whether the patient is hospitalized or recovering, individuals around the patient, including healthcare professionals, are less likely to offer advice on actively preparing for an RTW. Failure to discuss the timing of returning to work on an individualized basis can lower the patient's expectations of returning to work.

*S1: No one even asked me about going to work, I wish the nurses and doctors would tell us how far we have recovered before we can go back to work, and I don't know what kind of work is suitable after being sick.*

*S4: I took the initiative to ask about going to work, but my family said there was no hurry to earn money and asked me to recuperate for a while longer before considering this.*

*S9: Doctors usually give advice mainly on body diet and so on, without mentioning about work, so that means I'm not suitable to go to work at this stage either, right?*

#### 3.5.2 Accessing Employment and Job‐Seeking Information

Most patients indicated that they wanted to learn about job searching information, but they were unclear about how to obtain relevant information and could not fully utilize the Internet to find suitable positions.

*S2: Nowadays, information on the Internet is quite developed, but it is too abundant and chaotic. Even if I have the desire to work, I don't know where to find job‐seeking information.*

*S4: It would be helpful if we could receive timely information about suitable jobs. After I got sick, I was cut off from the news and unaware of changes in employment forms.*

*S7: I don't know how to look for a job on the Internet. As I get older, I find it difficult to use my smartphone.*

#### 3.5.3 Understanding Welfare Benefits and Legal Policies

Following their illness, patients frequently encounter various forms of injustice when reintegrating into the workplace. Some interviewees reported an inability to pursue effective legal recourse to safeguard their rights and interests after experiencing dismissal or demotion by their employers. This limitation was primarily attributed to a lack of sufficient knowledge regarding the legal protections and welfare provisions available to individuals who have experienced a stroke, as well as relevant labour laws.

*S5: It's not clear what benefit policies are in place specifically for people who have experienced a stroke.*

*S9: As soon as they heard I had this disease, the organization dismissed me immediately. Does the law address this? I don't know how to defend my rights.*

*S10: I'm still considered mid‐level leadership in my unit. Before my illness, the unit had me do my best in my role. However, after my illness, the leadership used my health as a reason to persuade me to move to a non‐leadership position. But I don't have the heart to care about this now.*

#### 3.5.4 Desire for Multi‐Faceted Assistance

Patients expressed the hope that society, the community, employers and healthcare institutions would collaborate to provide assistance in returning to work. They also emphasized the need for establishing links between institutions to achieve cross‐sectoral cooperation.

*S3: The leader of my unit doesn't understand this disease at all. He still thinks that after having this disease, the brain won't work well. I think we should have hospital personnel educate unit members about our physical condition, to show that we are still capable of working after getting sick.*

*S5: It would be beneficial to have an organization in the hospital that can assess the vocational skills of those of us who have been sick, to determine what kind of work we can still do. It would be even better if they could connect us with suitable jobs.*

*S11: I hope the state can improve laws and regulations to make it mandatory that employers are not allowed to discriminate against job seekers who have been sick.*

### 3.6 Personal Development Needs

#### 3.6.1 Pursuit of Self‐Actualization

Work holds multiple meanings for patients; it symbolizes recovery from illness and represents a return to normal life. Through work, patients can improve their sense of self‐identity and social value, feeling valued and needed, thereby realizing their own worth.

*S4: Being able to go back to work feels like a kind of spiritual support; at the very least, it means that my health is better.*

*S5: Being able to work means that I am a normal person. It reflects my value and helps me get my life back on track.*

*S6: In addition to being a means of earning a living, work helps me continue learning new knowledge, keep up with societal developments, and stay connected with society.*

#### 3.6.2 Development of New Skills

Patients who RTW after an illness face numerous challenges, including the need to learn new skills. Job searches often involve competition with healthy individuals, leading patients to worry that they will no longer be able to perform their jobs.

*S4: No one would want to hire someone who's been sick, right? I'm not competitive with anyone else, am I? Why would people choose me over healthy applicants?*

*S9: I used to drive a truck, but I can't drive anymore after I got sick. Now, at my age, it's hard to find a job, and I don't know what to study anymore.*

## 4 Discussion

### 4.1 Provide Continuous Rehabilitation Care Guidance to Improve Disease Management

This study found that patients' ability to perform daily activities was significantly impacted by their residual symptoms, which in turn reduced their work adaptability and confidence in returning to work. These findings are consistent with previous research [25]. However, the majority of patients exhibit a positive attitude towards returning to work and actively strive to re‐enter the workforce, demonstrating a strong need for effective self‐management of their condition throughout the process. Post‐stroke disease management requires comprehensive symptom monitoring and targeted guidance to enhance self‐management skills. This includes the use of memory aids, strategies for fatigue management and functional limb exercises [26].

Some patients encountered interruptions in their rehabilitation programmes due to insufficient continuity of care guidance following discharge or challenges in personal adherence. Prior research has demonstrated a high demand for continuity of care among individuals who have experienced a stroke, which includes comprehensive disease education, medical rehabilitation training and access to professional support through community resources [19, 27]. Continuity of care services enables patients to receive consistent, high‐quality rehabilitation guidance post‐discharge, crucial for correcting adverse emotions and behaviours and improving health outcomes [28]. Zhou et al. [29] found that self‐management programmes significantly enhance the social participation of stroke patients, particularly by encouraging active involvement in social, occupational and leisure activities. This, in turn, fosters greater independence and self‐confidence. These findings suggest that well‐designed self‐management programmes can help patients regain functionality in their daily lives, thereby improving their quality of life and reinforcing their social roles. Furthermore, Lo et al. [30] proposed a nurse‐led, community‐based self‐management programme aimed at supporting stroke patients in managing their rehabilitation process more effectively. By providing personalized health guidance, functional training and psychological support, this programme facilitates a smooth transition from the inpatient phase to community‐based rehabilitation, thereby preparing patients for a successful RTW. Thus, patients require comprehensive and continuous nursing guidance to enhance disease management. Healthcare professionals should provide regular, intensive education and management post‐discharge to maintain high levels of patient compliance [31].

In China, stroke patients face unique socio‐cultural and systemic challenges, including limited access to continuous rehabilitation services and varying levels of health literacy. developing and implementing culturally tailored self‐management programmes that address the specific needs of Chinese patients is of paramount importance. Such programmes can bridge gaps in rehabilitation services and significantly improve long‐term outcomes. Therefore, advancing the development and application of self‐management programmes in stroke rehabilitation—particularly those adapted to the cultural and systemic context of Chinese patients—has the potential to positively impact long‐term recovery and reintegration.

In this study, with a focus on the RTW process for stroke patients, there is increasing evidence highlighting the potential of integrative medicine in post‐stroke rehabilitation. Integrative medicine, which combines the strengths of both Western medicine and traditional Chinese medicine (TCM), has been shown to promote comprehensive rehabilitation through individualized treatment plans, thereby facilitating a successful RTW. Systematic reviews and meta‐analyses have demonstrated that the integration of Western and TCM approaches, particularly in motor function recovery, pain management and emotional regulation, can effectively assist patients in overcoming the physical and psychological challenges associated with returning to work [32]. Given that returning to work is a complex, multi‐stage process, further exploration of the application of integrative medicine is warranted to enhance the quality of care and long‐term rehabilitation services for stroke patients. This is particularly relevant in helping patients adapt to the work environment, improve their work capacity and manage occupational stress. investigating how integrative medicine can be more effectively incorporated into clinical practice may ultimately facilitate a smoother and more sustainable RTW journey for stroke survivors.

### 4.2 Foster a Supportive Social Environment to Enhance Emotional Support

The interview results indicated that patients seek support from multiple sources, and external social support can foster positive emotions and encourage individuals who have experienced a stroke to adopt a positive outlook, thereby facilitating their RTW, This aligns with prior research findings [33]. Given the prolonged nature of the rehabilitation process and the diverse emotional needs of stroke survivors, it is advisable to enhance the integration between patients and various support systems. This approach will maximize the benefits of external social support and increase patients' motivation to RTW. In the prolonged process of returning to work, Family members and friends should actively engage in the patient's preparation for returning to work by offering encouragement, supervision and reminders, helping the patient better navigate the challenges encountered during the process. Providing primary caregivers with a systematic understanding of post‐discharge care and guiding them to ensure the patient adheres to their rehabilitation exercise programme on schedule can significantly accelerate recovery and facilitate a return to normal life. Medical staff should maintain timely communication with patients to understand their internal needs and provide essential emotional support and assistance. Tailored to individual needs, healthcare professionals can deliver disease education, rehabilitation care and strategies for symptom management to both patients and caregivers, while establishing a multi‐platform care service model to enhance the continuity and effectiveness of care, promoting a smoother and more comprehensive recovery process.

Whilst the interview findings suggest that patients wish for employers to demonstrate understanding and support for stroke survivors by proactively adapting the work environment and workload or identifying suitable job roles, implementing these measures in practice can be challenging. This process often requires support from multiple sources, including greater awareness, organizational commitment, and access to external resources. To overcome these challenges, strategies such as targeted workplace education, training programmes and the development of support networks can help employers and colleagues better understand and address the needs of stroke survivors, fostering a more inclusive and supportive work environment [34]. Additionally, stroke survivors can benefit from peer support; establishing patient exchange meetings can offer a platform for collective emotional release, communication, peer encouragement, information sharing and the provision of role models from individuals who have successfully returned to work [35]. The state and society should also increase their attention to stroke survivors by enhancing public awareness and promoting the disease, thereby enabling patients to access more welfare resources and support through various public welfare platforms. Therefore, establishing a comprehensive social support network is essential to address the emotional support needs of patients and to assist them in returning to work more effectively.

### 4.3 Provide Abundant Information Support to Enhance the Ability to RTW

It was found through the interviews that the participants expressed a desire for information regarding retraining, such as the optimal time to RTW, retraining programmes, job search opportunities and legal and welfare policies, accessible through various channels. Currently, most domestic discharge guidance for patients recovering from stroke focuses on disease‐related health education and lacks vocational retraining content [36]. Given the individualized variations in information needs among stroke survivors, the internet represents an effective medium for acquiring information related to job searches, legal matters and welfare policies. However, considering that some patients may face challenges in utilizing digital technology, it is advisable to employ a combination of electronic and nonelectronic means [37]. Healthcare professionals can offer information support in various forms, including reinforcing verbal education, providing manuals on job search skills, evaluating the ability to RTW and offering guidance via video and telephone. Furthermore, inadequate communication and coordination among hospitals, rehabilitation units, employers, communities and social insurance companies have been identified as factors reducing the rate of patients' RTW. These stakeholders often lack understanding of patients' health conditions, making it challenging to accurately assess their ability and need to work, aligning with the findings of the interviews in this study [38]. Hence, it is essential for stakeholders to enhance cooperation to maximize the use of existing resources. This can be achieved by consolidating patients' illnesses and occupations for unemployment registration, establishing a comprehensive and systematic information platform for returning to work, regularly disseminating recruitment information and publicizing re‐employment policies and regulations. These efforts can help patients stay informed about the latest job opportunities and policies, enable stakeholders to understand patients' situations, and meet their diverse information needs. This approach facilitates the connection of information, management and relationships among hospitals, communities, families and employers.

### 4.4 Acquire New Job Skills to Enhance Self‐Worth

Most patients perceived work not merely as a means of earning a livelihood but as a platform for social engagement and a means of demonstrating their self‐worth, particularly for younger individuals. Some patients proactively sought to enhance their employability by acquiring new skills or exploring career paths better aligned with their post‐stroke capabilities. This often involved participating in vocational training, learning to utilize assistive technologies, or commencing with part‐time employment to gradually acclimate to the demands of the workplace. These findings suggest that stroke survivors view the process of returning to work as a pivotal step in re‐establishing their independence and self‐esteem, an enhanced sense of self‐worth appears to play a critical role in fostering their motivation to reintegrate into the workforce. Research indicates that involving people who have experienced a stroke in volunteer activities can be mutually beneficial for patients and service recipients [39]. This engagement can help patients gradually rebuild their work skills, develop teamwork and communication abilities, and prepare for full‐time or part‐time employment while also contributing to society and discovering their own value. However, implementing this practice necessitates addressing practical challenges, such as organizing volunteer opportunities, providing adequate training and encouraging patient participation. Additionally, negative public attitudes toward individuals who have experienced a stroke can adversely affect their willingness to RTW [25]. To counteract these issues, it is essential for the government, in collaboration with the media, to educate the public about the needs of stroke survivors. Efforts should be made to enhance understanding, shift public perceptions, foster a societal culture that is supportive and respectful of stroke survivors, and ensure equitable employment opportunities for them. Additionally, some patients may be unable to resume their previous jobs due to physical limitations post‐stroke, necessitating the acquisition of new skills. Community rehabilitation centres play a crucial role in this process. Communities should enhance their organization of employment guidance, allocate dedicated employment guidance personnel, offer diverse vocational rehabilitation skills training and provide tailored skills training courses for patients with varying needs. To further improve re‐entry rates, community support services can be integrated with medical rehabilitation centres to streamline services.

### 4.5 Limitations

This study has several limitations. First, the exclusion of individuals with aphasia limits the understanding of the specific needs of this group. Second, despite efforts to ensure sample diversity, a gender imbalance persisted, particularly with a lower representation of female participants, which may have influenced some of the study's conclusions. In traditional Chinese culture, men are typically viewed as the primary breadwinners, which may contribute to a higher likelihood of men returning to work after illness. Conversely, women are often expected to assume primary caregiving roles within the family, which may explain the underrepresentation of female participants in the study. Additionally, the perspectives of employers, family caregivers and healthcare professionals were not incorporated, which may have affected the comprehensiveness of our understanding of care needs. Finally, since the study was conducted in Zhengzhou, the capital city of Henan Province, the fast‐growing economy and high cost of living may exert considerable pressure on patients to RTW sooner. However, Zhengzhou's well‐established healthcare and rehabilitation resources may provide greater support, thereby enhancing patients' confidence and increasing the likelihood of their RTW.

## 5 Conclusion

The needs of young and middle‐aged individuals who have experienced a stroke in relation to returning to work are multifaceted, encompassing disease management, social support, access to RTW‐related information, personal development and other diverse forms of support. This underscores the necessity of improving communication and collaboration among stakeholders to offer comprehensive support to patients, thereby facilitating their RTW.

## Author Contributions

**Ziwei Liu:** conceptualization, investigation, writing–original draft, supervision, formal analysis. **Shu Liu:** writing–review and editing, funding acquisition, supervision, validation. **Jiaxing Shi:** supervision, validation, visualization, data curation. **Yanming Yang:** investigation, formal analysis, supervision. **Yuan Zhong:** formal analysis, data curation, supervision, investigation. **Jiaxin Li:** investigation, formal analysis, supervision.

## Conflicts of Interest

The authors declare no conflicts of interest.

## Data Availability

The data that support the findings of this study are available from the corresponding author upon reasonable request.
